# A conversation with W. Schuyler Jones, MD, Associate Professor of Medicine, Duke University

**DOI:** 10.1017/cts.2023.28

**Published:** 2023-03-20

**Authors:** Kathy Siranosian

**Affiliations:** Clinical Research Forum, Washington, DC, USA

## Top 10 Clinical Research Achievement Awards Q & A

This article is part of a series of interviews with recipients of Clinical Research Forum’s Top 10 Clinical Research Achievement Awards. This article is with W. Schuyler Jones, MD, Associate Professor of Medicine, Duke University. Dr. Jones is an interventional cardiologist with a specific focus on the diagnosis and treatment of patients with cardiovascular disease. Dr. Jones received a 2022 Top 10 Clinical Research Achievement Award for ADAPTABLE *(Aspirin Dosing: A Patient-Centric Trial Assessing Benefits and Long-Term Effectiveness)*. [1] *The interview has been edited for length and clarity.*

## What drew you to a career in clinical research?

My interest in clinical research started when I was a resident and continued during my fellowship at Duke University. By the time I joined the faculty here, it was clear to me that this is what I wanted to do. Cardiology is deeply rooted in clinical research. It’s how we can prove that a new therapy works or that known drugs or devices are safe and effective, and it’s crucial to what we do. That’s what drew me in.

## Your award-winning study has implications for cardiovascular care, as well as for large, pragmatic clinical trials, in general. Can you tell us more about how the trial began?

The aspirin question was asked about 20 years ago, but back then it wasn’t feasible to conduct a study across such a large population. Then in 2014, the Patient-Centered Outcomes Research Institute (PCORI) founded the National Patient-Centered Clinical Research Network (PCORnet), a “network of networks” to conduct comparative-effectiveness research. PCORI was looking for a relatively straightforward initial question to pose to the network, and after multiple levels of review, the question about aspirin regimens was selected. We designed the trial so that it would incorporate pragmatic methods and quality-by-design guiding principles to provide a real-world assessment of the comparative effectiveness of aspirin in routine cardiovascular care. We were also able to test the feasibility of leveraging new electronic methods to mitigate the burden of research on patients and sites and reduce total costs.

## So for this study, you were able to tap right into data that was already being collected?

That’s right—it’s part of what makes this trial so novel and interesting. PCORnet can collate electronic health record data and transform it into a common data model that can be utilized for research. There were 40 centers overall participating, and we used their collated data to measure outcomes. Being able to access data like this that has already been collected is really helpful and has enormous potential for future studies.

## That sounds like a highly collaborative approach

Yes, we engaged research partners at every step of the process. PCORI has helped shed light on the need and the desire for patient partnerships and patient-partnered research. This was my initial foray into that, and it was eye-opening in every sense and at every aspect of this study. We had nine patient partners who were representatives from many of the centers around the country, including some of whom had heart disease as well as some of whom participated in the study, and they had a chance to review the protocol to help with study activities. They were all on the steering committee, plus two were on the executive committee and two were on the data safety monitoring board. Even with simple questions like aspirin versus aspirin (81 mg vs 325 mg), patients want to know their clinician’s opinion, so we also partnered with clinicians because we knew it would take a broad collaborative effort to get this done. The framework was really for patient data and electronic health records information, but it turned into much more than that.

## Where is your research headed next?

I’m shifting gears from the aspirin question and working on a randomized controlled trial to learn if taking a statin could help older adults live well for longer by preventing dementia, disability, or heart disease. It’s called “PREVENTABLE (PRagmatic EValuation of evENTs And Benefits of Lipid-Lowering in oldEr adults)” and we’re using the same framework along with a partnership with VA hospitals around the country. We’ve randomized about 5,000 patients so far on our way to 20,000. These people will take a blinded study medication, and we’ll study cognitive impairment and dementia through annual phone calls and medical visits. We know that statins reduce the risk of cardiovascular events but this will be the biggest study ever of patients over 75 looking at how statins could improve cognitive impairment.

## What advice do you have for people who are considering a career in clinical research?

The great thing about clinical research is that it’s such a wide open opportunity. There are so many different levels of participation—from asking questions to a patient at a site to running big clinical studies. What it comes down to is staying curious. And the field is always changing. I think if anyone had asked us ten years ago if we could run a 15,000 patients study for, as it turned out, $17 million, we would have said “no.” But that’s what we did. That makes the work challenging, inspiring, and fun.

## Is that what motivates you?

What motivates me the most is the opportunity to change how we treat patients and ultimately, improve healthcare. That’s the overarching goal. It can be demanding but you have to keep the end goal in mind.

## What do you do to recharge, away from clinical research?

I have two teenagers, and so I end up spending most of my time with them when I’m outside the hospital or outside the research world. For the last 6 years, I’ve coached my son’s travel baseball team, and that’s become a really important outlet for me that isn’t medicine. You need something like that to put things in perspective and help you keep the focus on what’s important. I usually end up teaching the kids more about life than I do baseball because, well, now they’re 15, and I can’t teach them how to hit a curveball anymore.
